# Increased mesoscale diffusivity in response to acute glucose starvation

**DOI:** 10.17912/micropub.biology.000729

**Published:** 2023-02-21

**Authors:** Ying Xie, David Gresham, Liam J Holt

**Affiliations:** 1 Institute for Systems Genetics, New York University Langone Medical Center, New York, New York, United States; 2 Department of Biology, New York University, New York, New York, United States

## Abstract

Macromolecular crowding is an important property of cells that impacts multiple biological processes. Passive microrheology using single particle tracking is a powerful means of studying macromolecular crowding. Here we monitored the diffusivity of self-assembling fluorescent nanoparticles (μNS) and mRNPs ( *GFA1* -PP7) in response to acute glucose starvation. mRNP diffusivity was reduced upon glucose starvation as previously reported. By contrast, we observed increased diffusivity of μNS particles. Our results suggest that, upon glucose starvation, mRNP granule diffusivity may be reduced due to increased physical interactions, whereas macromolecular crowding in the cytoplasm may be globally reduced.

**Figure 1. The diffusivity of μNS particles increases upon acute glucose starvation, while the diffusivity of mRNP granules decreases.**
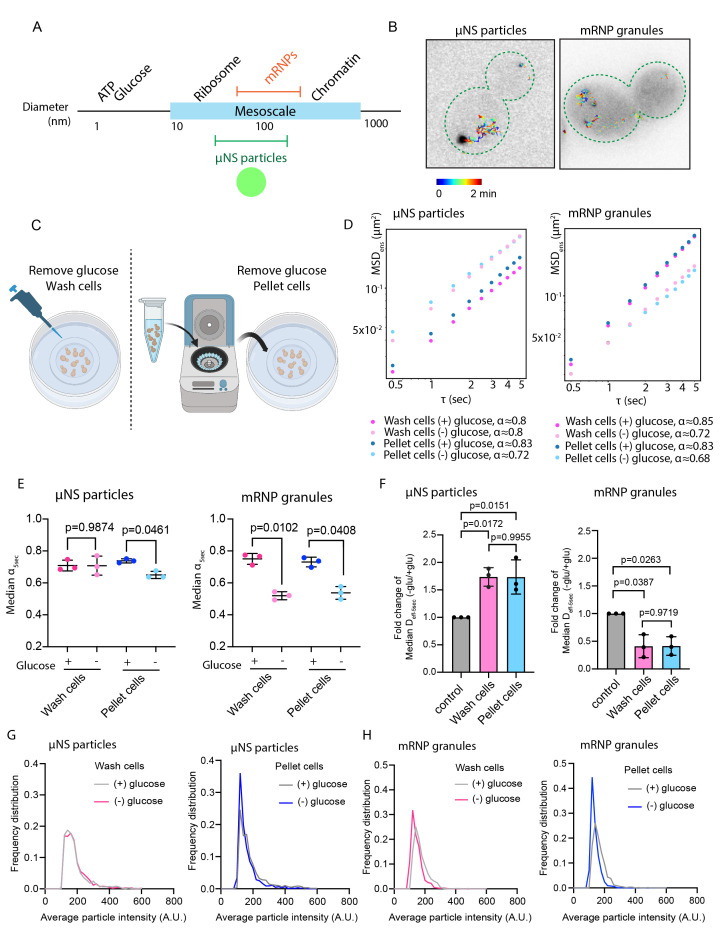
**A.)** Size of µNS and mRNP granules in relation to other macromolecules. **B.）** Representative time projections of µNS particles and mRNP granules using live cell imaging in *Saccharomyces cerevisiae* . **C.)** Two methods used to perform acute glucose starvation. **D.)** Ensemble-averaged mean-squared displacement (MSD) versus time delay (ꚍ), log10 scale. A linear model was fitted to determine the anomalous exponent α values for each condition at 30 min. (n = 700-1000 trajectories for µNS, n = 300-500 trajectories for mRNP granules) **E.)** Median anomalous exponent α-5sec from individual trajectory of three biological replicates. (mean ± S.D., P values were determined from a paired two-tailed t test). **F)** Fold change of median effective diffusion coefficients at 5 sec (D _eff-5sec_ ) from 3 biological replicate experiments (mean ± S.D., P values were determined from a paired two-tailed t test). **G-H)** Distribution of average particle intensities for µNS particles and mRNP granules cultured in the indicated media for 30 min. (n=700-1000 trajectories for µNS, n=300-500 trajectories for mRNP granules)

## Description

The cytoplasm is highly crowded as macromolecules such as RNA, protein and ribosomes occupy up to 30-40% of the cytoplasmic volume (Zimmerman and Trach 1991; Zhou, Rivas, and Minton 2008), leading to a phenomenon called macromolecular crowding. For simplicity, we will refer to macromolecular crowding as “crowding” henceforth. Crowding can influence biochemical reaction rates in several ways. First, high concentrations of crowders limit the excluded volume available to proteins or RNAs, thus impacting their thermal stability or proper folding (Pastore and Temussi 2022; Smith et al. 2015). Second, the crowded cytoplasm can favor intermolecular assembly, thereby accelerating reaction rates (Rivas and Minton 2018; Ellis 2001). Third, excessive crowding can dramatically decrease biomolecular diffusion rate, which slows down biochemical reactions (Rivas and Minton 2018; Ellis 2001). For example, various cellular processes can be affected due to the change of biomolecular diffusion rate. For instance, actin polymerization is accelerated by the addition of crowding agents (Drenckhahn and Pollard 1986), and transcription activity partly depends on how fast a transcription factor diffuses to gene loci (Berg, Winter, and von Hippel 1981). Therefore, changes in crowding can have profound effects on cell physiology.

The physical effect of crowding varies depending on the size of biomolecules. Length scales larger than individual macromolecular complexes such as ribosomes, but less than the size of the cell, define the mesoscale (~20 nm - 1 μm, (Ekman et al. 2017)). Many essential macromolecular complexes are of mesoscale size. DNA, RNA and proteins self-organize into a wide variety of structures such as mRNP granules and long range networks that are often highly dynamic ( **Figure 1A** ). Many mesoscale complexes are dynamically formed and remodeled depending on signals and environmental cues, and these processes typically involve active processes that consume energy (Sear, Pagonabarraga, and Flaus 2015). The biophysical principles behind tuning mesoscale complexes remain largely unknown. Modulating macromolecular crowding may be a mechanism for cells to regulate mesoscale complexes. For example, ribosomes are the main crowders in the cytoplasm and their concentration strongly affects the diffusivity of 40 nm particles. Manipulating ribosome concentrations through mTORC1 signaling has been shown to influence the phase separation behavior of synthetic condensates (Delarue et al. 2018).

Passive microrheology has been used in different studies to probe crowding levels and other cellular biophysical properties (Delarue et al. 2018; Shu et al., 2022; Parry et al. 2014; Munder et al. 2016; Etoc et al. 2018; Sabri et al. 2020). This method relies on analyzing the motion of tracer particles to infer the physical properties of a specific compartment. One such tracer particle, μNS, is derived from the mammalian orthorerovirus factory protein. The carboxyl-proximal regions (residues 471-721) of μNS fused with a GFP fluorescence tag can be easily expressed in cells through genetic engineering. μNS self-assembles into particles of varying size (50-150 nm) that are useful to probe mesoscale biophysical properties in the cell (Parry et al. 2014).

Prior work in bacteria showed that ATP depletion using toxins drastically reduces μNS particle diffusivity, suggesting that metabolic activity plays an important role in fluidizing the cytoplasm. Interestingly, this metabolic crisis showed a much stronger impact on the mobility of mesoscale particles > 50 nm in diameter, but had little effect on smaller particles (Parry et al. 2014). The motion of μNS particles has also been investigated in *Saccharomyces cerevisiae* (budding yeast) and *Schizosaccharomyces pombe* (Munder et al. 2016). Similar ATP depletion experiments in yeast cells balanced with different pH media suggests that acidification of the cytoplasm alters the biophysical properties of the cytosol. One possible explanation proposed by the study is that the acidified intracellular environment is close to the isoelectric point of a large portion of proteins, which leads to the formation of macromolecular assembly and therefore triggers the solid-like state transition of the cytoplasm. Interestingly, such solidification of cytoplasm correlates with a protective state for cell viability (Munder et al. 2016).

Chemically-induced ATP depletion is a relatively extreme scenario to challenge cell physiology. By contrast, microbes encounter frequent changes in nutritional status. For instance, glucose is an essential food source for budding yeast that can vary in abundance. Reorganization of intracellular structures and processes can be quickly and actively regulated upon nutrient perturbation coupled with the sudden change of physicochemical parameters, such as a drop in intracellular pH level, which was shown to signal the remodeling of the transcription program (Gutierrez et al. 2022; Triandafillou et al. 2020). Multiple processes including protein translation, endocytic trafficking, and inter-organelle communications are rapidly re-organized upon glucose starvation in yeast (Janapala, Preiss, and Shirokikh 2019; Laidlaw et al. 2021; Wood et al. 2020). Such reorganization is likely to affect the crowding level. However, it remains unclear how crowding changes at the mesoscale when cells are challenged by external stresses.

A study analyzing the mobility of cytosolic mRNP granules has suggested a reduction of mesoscale particle mobility upon acute glucose starvation (Joyner et al. 2016). One possible explanation proposed by this study is that reduction of the intracellular volume coupled with an increase of crowding reduces mesoscale motion. However, changes in crowding might not be the only explanation for reduced mRNP granule dynamics. We hypothesized that increased physical interactions between mRNP granules and intracellular components might also constrain mobility. Therefore, we compared mRNP granules to μNS particles to understand how endogenous and exogenous microrheology probes behave within the cytoplasm in response to acute glucose starvation ( **Figure 1B** ). Here, we studied one of the representative mRNP granules previously used (Joyner et al. 2016), the *GFA1* transcript, an essential gene involved in chitin biosynthesis. In brief, 24-PP7 stem-loops were integrated into the 3’ UTR of *GFA1* gene and the visualization of mRNPs was achieved by co-expressing the coat-binding protein, CP-PP7-3xYFP.

We also precisely controlled for several variables that are sometimes overlooked in acute glucose starvation experiments. First, crowding is sensitive to osmotic perturbation. The sudden removal of 2% glucose would reduce the osmotic pressure, leading to water influx and cell volume increase, thus complicating the interpretation of crowding changes. To avoid this, we osmotically balanced our starvation media with 2% sorbitol, which cannot be easily metabolized by budding yeast. Second, different groups have used different methods to switch cells to starvation medium. One method is to adhere cells on the imaging chamber and gently wash cells with starvation medium *in situ* . Another method is to use centrifugation to pellet then wash cells, typically 3-4 rounds of centrifugation/washing are used (Joyner et al. 2016). We were concerned that multiple centrifugation steps might contribute to additional compressive stress in cells (Peterson et al. 2012). As a result, we compared these methods to study both μNS particles and mRNP granules’ behavior upon acute glucose starvation ( **Figure 1C)** .

To quantify the changes of diffusivities of μNS particles and mRNP granules, we plotted ensemble-averaged mean square displacements (MSDs) versus time delay (τ), with fits to determine anomalous exponent α values ( **Figure 1D** ). We also calculated α values based on individual trajectories ( **Figure 1E** ). Both ensemble-time averaging analysis and individual trajectory analysis show consistent behavior for μNS particles and mRNP granules, displaying subdiffusive behavior (α < 1) ( **Figure 1D and 1E** ). The interpretation of the α value is complex but possible reasons include local caging effects or non-specific interactions between the particles and subcellular structures. Assuming the μNS particles do not have regulated interactions, the subdiffusive behavior in proliferating cells is likely due to the nonspecific interactions, and local caging from crowders or subcellular structures. Notably, glucose starvation significantly reduced the α value (p value < 0.05) for mRNP granules in both starvation methods ( **Figure 1E** ). Unlike μNS particles, the *GFA1* mRNP granule is an endogenous molecule, and therefore may be more likely to have specific increased physical interactions with cytosolic components in stress conditions.

We used hundreds of individual traces to quantify average displacement per unit time and computed effective diffusion coefficients at the 5 second timescale (D _eff-5sec_ ). A relative fold change of median D _eff-5sec_ in response to glucose starvation conditions to the median D _eff-5sec_ in proliferating cells was computed. Importantly, there was no obvious difference in our results for the two different methods used for inducing glucose starvation ( **Figure 1F** ). Consistent with the previous report (Joyner et al. 2016), a twofold decrease of diffusion rate was seen for mRNP granules ( **Figure 1F** ). By contrast, a 1.7 fold increase in diffusion rate was observed for μNS particles ( **Figure 1F** ). Changes in μNS particle sizes are a possible explanation for the observed increase in effective diffusion coefficient. To test this possibility, we quantified the average intensity of particles as a proxy for particle size (Parry et al. 2014), and compared the average particle intensity distribution before and after glucose starvation ( **Figure 1G and 1H** ). For μNS particles, the particle intensity distributions were similar before and after starvation ( **Figure 1G** ), suggesting the decreased particle diffusivity was not due to changes in particle size. On the other hand, we observed a slight overall reduction in average particle intensity of mRNP granules upon glucose starvation regardless of the experimental methods ( **Figure 1H** ). This observation rules out the possibility that increased particle sizes explains the observed reduction in mRNP granules diffusivity.

Our study identified contrasting behavior of two different microrheology probes upon acute glucose starvation. A previous report has suggested caution in the interpretation of mRNP granule dynamics when using the PP7 stem–loops (PP7SL) system in budding yeast cells. Comparison with single-molecule fluorescence in situ hybridization (smFISH) revealed that engineering stem-loops into the *GFA1* 3’ UTR caused aberrant sequestration of the 3′-end bacteriophage stem-loops fragments, but not the gene-specific mRNA moiety fragments, into processing bodies upon glucose starvation, suggesting PP7 stem–loop labeling in general affects the degradation and/or localization of mRNAs (Heinrich et al. 2017). Thus, it is possible that physical interactions of *GFA1-PP7SL* transcripts with RNA binding proteins in the processing bodies constrain diffusivity, leading to the observed reduction in D _eff-5sec_ of mRNP granules upon acute glucose starvation. On the other hand, an increase of D _eff_ for μNS particles upon acute glucose starvation could be due to several reasons: 1) a reduction of crowder numbers; 2) a remodeling of crowders may globally reduce the confinement of mesoscale macromolecules; or, 3) an increase in non-thermal energy may promote diffusivity in the cell. However, since ATP levels are decreased upon glucose starvation (Joyner et al. 2016), we do not favor this latter hypothesis. The contrasting changes of D _eff_ from μNS particles and mRNP granules highlight the importance of utilizing different foreign tracer probes to study the biophysical properties of the cell.

The sudden removal of glucose to starve yeast cells represents a distinct physiological change compared to yeast cells that approach saturation in laboratory culture medium. We argue that both scenarios are interesting and biologically relevant. For example, in natural environments yeast cells can easily be washed off of nutrient-rich surfaces by rain leading to instant starvation, or they could grow until they deplete all nutrients *in situ* . In the latter case, cells are able to accumulate glycogen and trehalose when glucose is gradually diminished, but these storage carbohydrates do not accumulate upon acute glucose starvation. Glycogen and trehalose have been shown to affect microviscosity and decrease the mobility of both nanoscale protein molecules (GFP at 3 nm diameter) and the diffusivity of particles at the mesoscale of 500 nm (Persson, Ambati, and Brandman 2020).

In conclusion, our observations with µNS particles show that unknown cellular adaptation mechanisms can increase mesoscale particle diffusivity upon acute carbon starvation. It will be interesting to further explore and understand possible mechanisms for this physical change.

## Methods

Table 1. Yeast strains used in this study

**Table:** 

strain	source	Identifier
W303 *MATa leu2-3,112 trp1-1 can1-100 ura3-1 Ade+ his3-11,15, pHIS3-GFP-µNS (1413 to 2160 n.t.) -URA3*	Holt lab	LH3407
W303 *MATa/* S288c *MATα NDC1/ndc1::NDC1-tdTomato::KANMX GFA1/gfa1::GFA1-24PP7 3xYFP-PP7-CFP::HIS3*	(Joyner et al., 2016)	KWY4736

**Yeast culture**

*Saccharomyces cerevisiae* strains used in this study are listed in Table 1. Strains were revived from -80°C freezer on YPD plate for an overnight growth, and then a patch of cells were inoculated in synthetic complete media + 2% dextrose (SCD) according to standard Cold Spring Harbor Protocols (“Synthetic Complete (SC) Medium,” 2016). The cultures were grown at 30°C in a rotating incubator for 4-5 hours without exceeding an O.D.600 of 0.4. Afterwards, the cultures were diluted for an overnight growth in order to reach O.D.600 between 0.1 and 0.4 for the next day's imaging experiment.

**Acute glucose starvation**

Switching cells to starvation medium without pelleting by centrifugation: Cells with culture medium reaching O.D.600 = 0.1–0.4 were applied to imaging chamber, which was pre-coated by 1mg/ml concanavalin A to immobilize cells on the coverslip. After waiting for 5-10 min for cells to settle down on the coverslip, the culture medium was removed completely, and four additional wash of cells with SCD or SC medium supplemented with 2% sorbitol (to balance the osmotic pressure) was performed. Cells were imaged at 30 min afterwards.

Switching cells to starvation medium by centrifugation: 1 ml cells grown in SCD media (O.D.600 = 0.1–0.4) were collected by centrifugation (3000 rpm) for 2 min. The supernatant was removed and cells resuspended in 1 ml of SC with 100 mM sorbitol media. Four additional wash steps followed, with two 2 min spins (6000 rpm) succeeded by two 1 min spins. The cells were suspended into fresh SC+2% sorbitol or SCD media for analysis at 30 min.

**Imaging and particle tracking**

Single particle tracking in *Saccharomyces cerevisiae* was performed for the µNS particles and GFA1-mRNP granules. The particles were imaged using Andor Yokogawa CSU-X confocal spinning disc on a Nikon Ti2 Eclipse microscope and fluorescence was recorded with a sCMOS Prime 95B camera (Photometrics) with a 63x objective (pixel size: 0.18 μm), at a 500 ms image capture rate , with a time step for 2 min. The tracking of particles was performed with the Mosaic suite of FIJI (Sbalzarini & Koumoutsakos, 2005), using the following typical parameters: radius = 2; cutoff = 0; percent: variable, a link range of 1, and a maximum displacement of 5 pixels, assuming Brownian dynamics.

**Quantification of mesoscale rheology**

For every trajectory, the time-averaged mean-square displacement (MSD) was quantified as defined in (Delarue et al., 2018; Joyner et al., 2016; Munder et al., 2016; Shu et al., 2022). To characterize individual particle trajectories, we calculated effective diffusion coefficients by fitting MSD with a linear (diffusive) time dependence within ten time points. Particle trajectories with more than ten time points were selected for analysis to reduce tracking error. Time-averaged MSD for each trajectory is fitted using a linear time dependence over the first ten time intervals:


MSD(τ)=4Deffτ

where D _eff_ is the effective diffusion coefficient for each trajectory, τ is the time delay. And we refer to the median value D _eff_ from all the trajectories at each condition to represent its effective diffusion coefficient.

Ensemble-time averaged MSD was applied for better indication of anomalous exponent α at each condition. which is ensemble-averaged from all time-averaged MSD for trajectories with more than ten time points:


$$MSD(\tau )T-ens=4D\tau^{\alpha }$$

To measure the mean intensity of particles, a fixed radius (radius=3) was used along the movie series, and the mean intensities of particles were measured at all the tracked frames and then summarized as average mean intensity.

All quantifications were performed in the programme GEMspa (https://github.com/liamholtlab/GEMspa) and graph plots were generated in GraphPad Prism 9.
